# Speech identification and cortical potentials in individuals with auditory neuropathy

**DOI:** 10.1186/1744-9081-4-15

**Published:** 2008-03-31

**Authors:** Vijaya kumar Narne, CS Vanaja

**Affiliations:** 1 Department of Audiology, All India Institute of Speech and Hearing, Mysore, Karnataka, 570006, India; 2 Bharati Vidyapeeth University School of Audiology and Speech Language Pathology, Dhankawadi, Pune, Maharashtra, 410021, India

## Abstract

**Background:**

Present study investigated the relationship between speech identification scores in quiet and parameters of cortical potentials (latency of P1, N1, and P2; and amplitude of N1/P2) in individuals with auditory neuropathy.

**Methods:**

Ten individuals with auditory neuropathy (five males and five females) and ten individuals with normal hearing in the age range of 12 to 39 yr participated in the study. Speech identification ability was assessed for bi-syllabic words and cortical potentials were recorded for click stimuli.

**Results:**

Results revealed that in individuals with auditory neuropathy, speech identification scores were significantly poorer than that of individuals with normal hearing. Individuals with auditory neuropathy were further classified into two groups, Good Performers and Poor Performers based on their speech identification scores. It was observed that the mean amplitude of N1/P2 of Poor Performers was significantly lower than that of Good Performers and those with normal hearing. There was no significant effect of group on the latency of the peaks. Speech identification scores showed a good correlation with the amplitude of cortical potentials (N1/P2 complex) but did not show a significant correlation with the latency of cortical potentials.

**Conclusion:**

Results of the present study suggests that measuring the cortical potentials may offer a means for predicting perceptual skills in individuals with auditory neuropathy.

## Background

Auditory neuropathy is one of the hearing disorders in which cochlear amplification is normal but neural transmission in afferent pathway is disordered. The integrity of cochlear function in this population is provided by the presence of evoked oto-acoustic emissions and/or cochlear microphonics (CM), and the abnormal neural transmission or dys-synchrony is indicated by the absence of auditory brainstem responses and middle ear muscle reflexes. Although the audiological findings in auditory neuropathy are suggestive of a retro-cochlear pathology, the exact site of pathology and patho-physiological mechanism leading to auditory neuropathy is not known. Two physiological explanations proposed for the neurophysiological manifestations observed include dys-synchronized spikes discharge and/or reduced spike of the auditory nerves [1,2].

Hearing sensitivity in individuals with auditory neuropathy may range from normal hearing to profound hearing impairment [3]. A majority of individuals with auditory neuropathy have low frequency hearing loss with disproportionately poor speech recognition scores for the degree of hearing loss [3]. Speech identification ability in individuals with auditory neuropathy varies considerably among patients but approximately 60 to 70% of individuals have identification scores well below the estimated identification scores from their pure-tone thresholds [3,4].

Starr et al. [5] attempted to record click evoked cortical potentials (P1, N1 and P2) in four of ten adults subjects with auditory neuropathy. The responses to supra-threshold click stimuli were recordable in three of four subjects. They further observed that the subject with absent cortical potentials had poorer speech identification score than other three subjects. Kraus et al. [6] subsequently presented a case report showing cortical evoked potentials in a teenager with auditory neuropathy, whose identification score in quiet was 100%, whereas in adverse conditions, the identification scores were very poor. As the cortical potentials were normal in this client, they hypothesized that speech perception in quiet was not significantly affected by poor synchronization at the brainstem level if synchronization is preserved at the cortical level. Results of some of the investigations carried out later support this hypothesis. Rance et al. [7] observed better speech identification scores in children with auditory neuropathy who had normal cortical potentials when compared to those with abnormal cortical potentials. Vanaja and Manjula [8] reported that individuals who have higher amplitude in cortical potentials had better speech identification scores and also benefitted more with a hearing aid than those with lesser amplitude.

Thus, limited information available in literature shows that auditory neuropathy individuals having poor identification scores in quiet have abnormal or absent cortical potentials suggesting that integrity of processing at cortical level is important for speech understanding. The present study was undertaken to study the relationship between speech perception ability in quiet and parameters of cortical potentials in individuals with auditory neuropathy.

## Methods

### I. Participants

Ten individuals with auditory neuropathy and ten individuals with normal hearing participated in the present study. Out of ten individuals with normal hearing, five were males and five were females with ages ranging from 12 to 39 yr with a mean of 22 yr. The individuals with normal hearing had pure-tone sensitivity of less than 15 dB HL at octave frequencies from 250 Hz to 8000 Hz. These individuals were volunteers from local college and schools.

Participants with auditory neuropathy were recruited from the clients registered at the Audiology clinic of the All India Institute of Speech and Hearing, Mysore, India. Table 1 shows the audiological profile of the ten participants (5 males and 5 females) with auditory neuropathy. The age of the participants ranged from 12 to 39 yr with a mean of 20.7 yr. The pure-tone average (average of pure tone thresholds at 500, 1000, 2000, 4000 and 8000 Hz) ranged from 10 to 48 dB HL. A majority of the participants had symmetrical hearing loss in both the ears. The audiometric configuration was rising pattern in a majority of the participants. All the participants had present TEOAEs and absent middle ear acoustic reflexes (both ipsilateral and contralateral) and the auditory brainstem responses. None of participants had any family history or any other medical complications. All the participants were native speakers of Kannada, a Dravidian language spoken in a southern state of India.

**Table 1:** Audiological profile of individuals with auditory neuropathy

S.No	Age/Sex	Pure-tone Average (dB HL) Right ear	Pure-tone Average (dB HL) Left ear	ABR in both ears	OAE in both ears	Acoustic reflex in both ears
AN1	12 ys/M	26.00	31.00	Absent	Present	Absent
AN2	20 ys/F	31.00	34.00	Absent	Present	Absent
AN3	15 ys/F	30.00	36.00	Absent	Present	Absent
AN4	39 ys/F	33.00	39.00	Absent	Present	Absent
AN5	12 ys/M	44.00	43.00	Absent	Present	Absent
AN6	24 ys/M	31.00	38.00	Absent	Present	Absent
AN7	27 yr/F	42.00	31.00	Absent	Present	Absent
AN8	20 yr/M	48.00	46.00	Absent	Present	Absent
AN9	18 yrs/M	19.00	10.00	Absent	Present	Absent
AN10	20 yrs/M	43.00	39.00	Absent	Present	Absent

### II. Data Collection

#### a. Assessment of speech identification ability

##### Stimuli

Speech Identification Test in Kannada developed by Vandana [9] was used to assess open set speech identification abilities. This test consists of 50 bi-syllabic meaningful words of Kannada. Validity and reliability of this test has been established on native speakers of Kannada [9].

##### Procedure

The participants listened to speech tokens individually in a double-walled, acoustically treated room where the ambient levels were within permissible limits [10]. The speech stimuli were presented through supra-aural headphones (TDH – 39) of a calibrated [11] diagnostic audiometer (Madson OB-922). The stimuli were presented at 40 dB SL (re: Speech Recognition Threshold) monaurally and the participants were asked to repeat the speech token. The speech recognition scores were calculated by counting the number of words correctly repeated.

#### b. Cortical evoked potentials

The participants were seated comfortably in a reclining chair and the cortical evoked potentials were acquired using the Intelligent Hearing Smart EP system. The responses were picked up from a disc electrode placed on the midline site, Cz, with reference to an electrode placed on the ipsilateral mastoid. The common electrode was placed at Fpz. It was ensured that the impedance at each electrode site was less than 5 k ohms and the inter-electrode impedance was less than 2 k ohms. The participants were instructed not to pay attention to the stimuli while recording.

The cortical potentials were recorded for each ear separately with click stimuli presented through insert-earphones (ER-3A) at a repetition rate of 1.1/sec at 80 dB nHL. Stimulus level used to elicit the cortical waveforms were supra-threshold for all participants. The EEG acquired was amplified 50,000 times and digitally filtered using a band pass filter of 1–30 Hz. The EEG was epoched using a window of 550 ms, including a 50 ms pre-stimulus baseline. Epochs greater than 45 μV were rejected. The EEG responses for 200 stimuli were averaged. The latency of P1, N1, P2, N2, and the amplitude of N1/P2 were measured. The amplitude of N1/P2 was measured with peak-to-peak.

Recordings were repeated twice to check for replicability. Only those peaks, which were replicable, were considered as a response. Three experienced audiologists independently analyzed the waveforms to identify and mark the peaks in cortical potentials. It was considered as a response only if all the three audiologists identified the cortical potentials at the same latency.

## Results

### Speech identification ability

**Figure 1:**
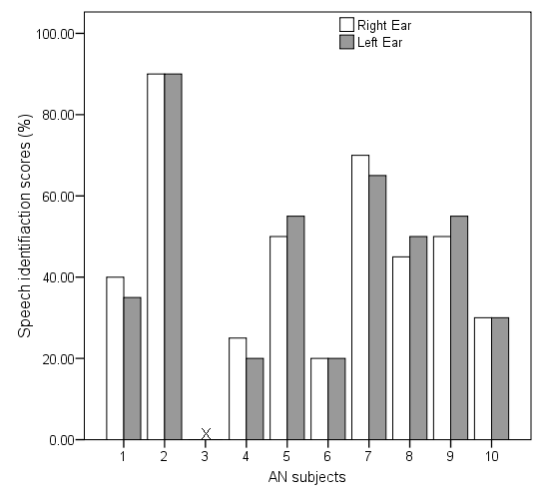
**Speech identification scores of the individuals with auditory neuropathy.** In the figure, X indicates of 0% identification scores.

Speech identification scores in individuals with normal hearing ranged from 95% to 100% with a mean of 96% in both eras whereas in individuals with auditory neuropathy identification scores in both eras ranged from 0 to 90% with a mean of 42.1% in the right ear and 41.2% in the left ear. Among the individuals with auditory neuropathy, AN-3 had 0% identification in both eras. Figure 1 shows the individual data for speech identification scores in individuals with auditory neuropathy. Paired sample "t" test revealed no significant difference (auditory neuropathy: t = 0.1, p = 0.88; Normal: t = 0.05, p = 0.9) between the ears for identification scores in both groups. Hence, the data from the two ears were merged for further statistical analysis.

The mean speech identification scores for subjects in the normal hearing group was 96% with a standard deviation of 2.5% whereas the mean scores of individuals with auditory neuropathy was 42% with a standard deviation of 25.4%. An Independent Sample't' test revealed a significant difference between the mean speech identification scores of the two groups (t = 5.77, p < 0.01).

**Figure 2:**
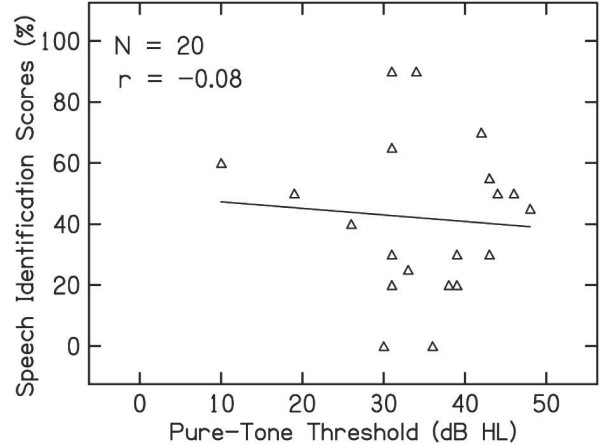
Relationship between the pure-tone threshold and identification scores of individuals with auditory neuropathy.

Pearson product-moment correlation was performed between behavioral threshold and speech identification scores in individuals with auditory neuropathy. Figure 2 shows the scatter plot between pure-tone average and speech identification scores. Pearson correlation coefficient revealed that there was no significant correlation between speech identification scores and pure-tone average in individuals with auditory neuropathy (r = -0.37, p = 0.6).

### Cortical potentials

Cortical evoked potentials were present and symmetrical in all the individuals with normal hearing. Cortical potentials were present and symmetrical in all the individuals with auditory neuropathy, except one participant (AN3). The responses were absent in a 15 year old participant with a pure-tone average of 30 dB HL. Therefore, the age and threshold cannot be the contributing factors for the absence of responses in this participant.

Paired Sample" t" test was performed to compare between two ears for latency of cortical potentials (P1, N1, P2 and N2) and amplitude of N1/P2. The results revealed no significant difference between the two ears. For further analysis, data of right ear and left ear were combined. The mean and standard deviation of latencies of P1, N1, P2, N2 in individuals with normal hearing and those with auditory neuropathy are presented in Table 2. From the table it can be noted that the latencies in subjects with auditory neuropathy were delayed by 20 – 50 ms for P1, 40–80 ms for N1 and 30–80 ms for P2 when compared to individuals with normal hearing.

**Table 2:** Mean, SD, and "t" value of latencies and amplitude cortical potentials in individuals with normal hearing and auditory neuropathy

Participants	Latencies (m sec)				Amplitude N1/P2 (μV)
		
	P1	N1	P2	N2	
AN	76 (20)	124(31)	185(43)	243(50)	4.4(2.4)
Normal	50 (8.1)	85(9)	142(12)	218(13)	6.2(1.3)
"t" Value	4.1*	2.8*	3.05*	1.6	- 0.82
"p" Value	0.001	0.001	0.001	0.057	0.05

* Significant at p < 0.01

Independent sample "t"test was performed independently for latency of cortical potentials (P1, N1, P2 and N2) and amplitude (N1/P2). Results revealed a statistically significant difference between the latencies of P1, N1, and P2 peaks in individuals with normal hearing and those with auditory neuropathy but there was no significant difference for the latency of N2 peak. The "t" value and the level of significance are also shown in Table 2. The N1/P2 amplitude of the participants with auditory neuropathy did not differ significantly from that of normal hearing individuals. However, the mean values of the amplitude for the participants with auditory neuropathy were slightly lower and the variability was greater when compared to those observed in normal subjects.

As there was more variability in measures of individuals with AN, the data of the participants with auditory neuropathy were further divided into two groups based on their speech identification scores. Group I included "Good Performers" whose speech identification score was more than 50% and Group II included "Poor Performers" whose speech identification score was less than or equal to 50%. The mean and standard deviation of latency and amplitude (N1/P2) cortical potentials for the two groups are presented in Table 3. It can be noted that the amplitude of Poor Performers was lower than that of Good Performers. Results of Kruskal Wallis test revealed that there is a significant effect of group on the amplitude (p < 0.01) of N1/P2 peak. Mann-Whitney test was performed to assess the paired comparison between the groups. Results revealed that the mean amplitude of Poor Performers was significantly lower than that of Good Performers (p < 0.01) and normal hearing subjects (p < 0.01). However, mean amplitude of Good Performers was not significantly different from that of normal hearing subjects (p > 0.01).

**Table 3:** Mean and SD of latencies and amplitude cortical potentials for the two groups of auditory neuropathy and individuals with normal hearing

Participants	Latency (msec)				Amplitude
	P1	N1	P2	N2	N1/P2 (μV)

Normal	50 (8.1)	85(9)	142(12)	218(13)	6.2(1.3)
Good Performers	84(16.8)	133(30.8)	186(53.8)	227(26.2)	6.0(1.5)
Poor Performers	78(20.2)	125(27.6)	184(27.4)	231(18.2)	2.6(1.3)

Kruskal Wallis test performed to study the effect of group on the latency of cortical potentials revealed that there was a significant effect of group on the latency (p < 0.01) for all the components except for N2. Mann-Whitney test was performed to assess the paired comparison between the groups. Results revealed that both in Good Performers and Poor Performers, the mean latency for all the peaks except N2 differed significantly from that of normal hearing subjects but there was no significant difference for latency for all the peaks between Good Performers and Poor Performers (p < 0.01).

**Figure 3:**
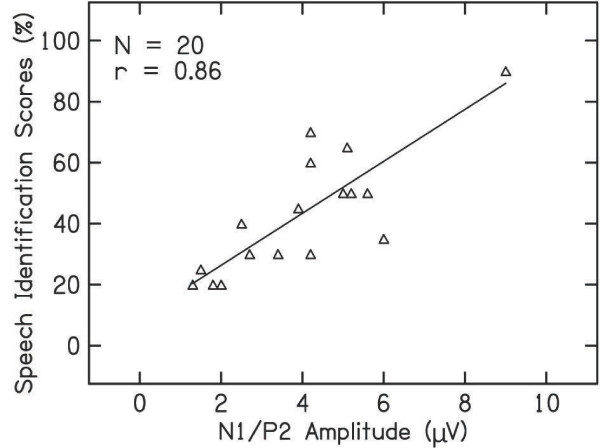
Scatter plot between speech identification scores and amplitude of cortical potentials in individuals with auditory neuropathy.

Pearson product-moment correlation was carried out to study the correlation of the peak latency of P1, N1, P2, N2 and the amplitude of N1/P2 with the behavioral thresholds (pure-tone average) and speech identification scores. It can be observed from Table 4 that the latency of cortical potentials did not show a significant correlation with the pure tone average or with speech identification scores. However, the amplitude of N1/P2 showed a significant correlation with speech identification scores. Relation between N1/P2 amplitude and speech identification scores is depicted in the scatter plot along with regression curve in Figure 3.

**Table 4:** Correlation coefficients (r) of behavioral thresholds with cortical potentials and word recognition scores with cortical potentials

	*"r" value*				
	
	Latency *(ms)*				N1/P2
	P1	N1	P2	N2	amplitude (μV)
Behavioral Threshold	-0.46	-0.25	-0.2	-0.3	-0.16
Speech Identification score	0.45	0.52	0.3	-0.1	0.86*

* Significant at p < 0.05

**Figure 4:**
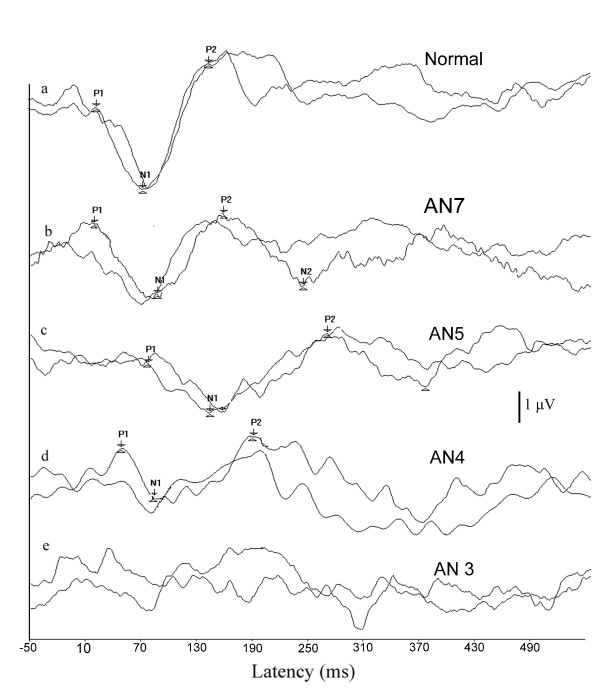
**Representative samples of cortical potentials recorded from the two groups.** a) Wave forms of individuals with normal hearing. b) Wave forms with normal latencies and normal amplitude obtained from an individual with auditory neuropathy. c) Wave forms with prolonged latencies obtained from an individual with auditory neuropathy. d) Wave forms with normal latency and reduced amplitude obtained from an individuals with auditory neuropathy e) No response obtained from an individual with auditory neuropathy.

Inspection of individuals data revealed that two participants had normal latencies with reduced amplitudes and their speech identification scores were poor (AN4, AN6), whereas two participants who had normal latencies with good amplitude (AN2 and AN7) had better speech identification scores. Five subjects who had prolonged latencies with normal amplitude also showed good speech identification scores. Figure 4 shows the waveforms of individuals with auditory neuropathy and those with normal hearing.

## Discussion

### Speech identification in individuals with auditory neuropathy

In the present study, speech identification scores in individuals with auditory neuropathy were significantly lower than that observed for participants with normal hearing. Further, the correlation analysis revealed that speech identification scores for individuals with auditory neuropathy were disproportionate to their pure-tone threshold. This is best illustrated by comparing the speech identification scores in individuals with auditory neuropathy to those expected by degree of hearing loss for patients with cochlear hearing loss [12]. In the present study, in 72% of individuals, the speech identification scores were lower when compared to those reported by Vanaja and Jayaram [12] for ears with sensorineural hearing loss. Sininger and Oba [3] observed that speech identification scores for 69% of their patients with auditory neuropathy were lower than that reported for patients with cochlear pathology by Yellin et al. [13]. These results suggest that speech identification scores do not depend upon the pure-tone thresholds in individuals with auditory neuropathy. Other factors impair the speech understanding capability in these individuals.

One of the possible contributors for their poor speech identification score is disrupted neural synchrony, which impairs the listener's ability to processes the dynamic nature of speech signals. It has been reported that disrupted neural synchrony impairs the ability to use envelope cues in speech and also impair the ability to perceive rapid change of spectral shapes in the speech stimuli [2,4,14].

### Cortical potentials in individuals with auditory neuropathy

Latencies of cortical potentials in individuals with auditory neuropathy were significantly prolonged when compared to normal hearing listeners. Though not statistically significant, the mean amplitude of the cortical potentials was lower than that observed in participants with normal hearing and the variability was high in individuals with auditory neuropathy. Latencies and amplitude variations in individuals with auditory neuropathy may not be due to increased pure-tone threshold, as there was no correlation between pure-tone thresholds and cortical potentials (latency and amplitude), suggesting that the latency and amplitude of cortical potentials were not affected by the hearing thresholds of the participants in the present study. Oates, Kurtzberg, and Satpells [15] reported that the latencies and amplitude of P1/N1/P2 were not significantly affected in subjects with cochlear hearing loss of less than moderate degree. Cortical potentials were absent in AN3 who had pure-tone threshold of 30 dB HL whereas it was present in AN 4 whose pure-tone average of 48 dB HL. This further, supports the notion that cortical potentials did not depend upon the pure-tone average in the present study.

Cortical potentials mature and attain adult latency and morphology by the age of 9 years and there will not be any significant changes in latency until age of 50 yr [16]. As the age range of the participants in the present study varied from 12 to 39 yr, the latency variations observed may not be due to maturational changes. It was hypothesized that probably the severity of the neural dys-synchrony rather than the hearing loss contributed for the variability in the cortical evoked responses observed in the present study.

An interesting observation in the present study was that, some of individuals with auditory neuropathy had abnormal latencies with normal amplitude, whereas some had normal latencies with abnormal amplitude in cortical potentials. Similar results have also been reported in the literature [5,8,17]. No clear-cut explanation can be provided for the variability observed in latencies of cortical potentials. The variability in latencies observed across individuals with auditory neuropathy in the present study may have been related to the underlining patho-physiology. That is prolonged latencies may be due to the dys-synchronous firing [18-20,14] whereas normal latencies may be due to the reduced numbers of fibers [1,14]. The magnitude of reduction in amplitude in either of patho-physiology depends upon the severity of the condition [14,21]. Further investigation correlating cortical potentials with neurological findings need to be carried out to confirm this.

### Relation between speech identification scores and cortical potentials

In the present study individuals who had cortical potentials with better amplitude had better speech identification scores than those with absent/abnormal amplitude in cortical potentials. Participant AN3 in the present study had absent cortical potentials and very poor speech identification scores. Similar results have been reported by earlier investigators [7,5,17]. These results suggest that it is possible to have good speech perception in quiet if the cortical responses are present even if the brainstem responses are abnormal. Good synchronization at the auditory nerve and brainstem level does not appear to be essential for understanding speech in quiet situations [6]. Results of physiological studies indicate that brainstem neurons process the fast modulations of the complex signals, whereas auditory cortex processes the slowly varying the amplitude modulations of the complex signal [22], which plays an important role in auditory communication [23].

There was also a high positive correlation between speech identification and the amplitude of N1/P2. That is, individuals with better speech identification scores showed greater N1/P2 amplitude than those with poorer speech identification scores. However, no correlation was observed between latencies of cortical potentials and speech identification scores. Similar findings were observed in adults [8] and children[7] with auditory neuropathy using hearing aids. They reported that the cortical potentials were present in individuals with auditory neuropathy who had good speech identification scores and these individuals also benefited from a hearing aid. Thus, it can be concluded that the speech recognition scores probably depend on the severity of the disorder rather than the underlining patho-physiology.

## Conclusion

Results of the present study support the previously reported findings that speech perception ability cannot be reliably estimated from behavioral pure-tone audiogram in individuals with auditory neuropathy. Cortical potential testing may, however, offer a means of predicting speech understanding ability in individuals with auditory neuropathy. The presence and amplitude of cortical potentials showed a significant correlation with open set speech perception abilities. The absence of cortical potentials indicates extremely poor speech perception abilities. If these results are replicated in a larger group of individuals with auditory neuropathy, the procedure can be used to obtain important information regarding severity and management options for these participants.

## Authors' contributions

VKN was involved in designing the study, data collection, analysis, interpretation of results and preparing the manuscript. CSV was involved in designing the study, analysis interpretation of results, and preparing the manuscript.
